# Scraps

**Published:** 1901-12

**Authors:** 


### SCRAPS

PICKED UP BY L. M. G.

**The Doctor's Fee.**—In a paper bearing this title which Dr. John B. Roberts read before the Philadelphia County Medical Society, he makes from his personal experiences some alarming revelations concerning the commercial instinct of medical men. The *Medical News* of June 8th, commenting on the paper, says that to the professional vices mentioned by Dr. Roberts should be added those of advertising and exaggeration, and it gives instances of these to which reference should be made because the temptation to fall into some of these evil ways might come to somebody on this side of the Atlantic.

**Malapropisms.**—From a recent number of *Truth* I take these, which have a medical interest:—

"The sewage of St. Albans having created a great nuisance in the village of Park-street, certain of the inhabitants were getting up a memorial to the Corporation to abate the nuisance. An old woman declared that at first she could not abide the smell, but now she was getting manured to it."

"A little girl, writing a letter to her governess absent on holiday, wrote: 'Dear Miss Brown, I wish you posterity in the coming year.'"

"An old lady said that the War Office had neglected her son because he had very coarse veins."

**A Doubtful Case.**—"We hear from Taunton that at a General Quarter Sessions of the Peace for the County of Somerset held there lately, Mary Hamilton, otherwise George, otherwise Charles Hamilton was try'd for a singular and notorious offence. The Council for the King open'd to the Court. That the said Mary, etc., pretending herself a Man had married FOURTEEN WIVES, the last of which number was one Mary Price, who appeared in Court, and deposed, that she was married to the Prisoner, some little Time since, at the Parish Church of St. Cuthbert's, in Wells, and that they were Bedded as Man and Wife, and lived as such for about a Quarter of a Year, during which Time she, the said Price, thought the Prisoner a Man owing to the Prisoner's using certain deceitful Practices not fit to be mentioned.

"There was a great Debate for some Time in Court about the Nature of her Crime, and what to call it, but at last it was agreed, that she was an uncommon Notorious Cheat, and as such was sentenced to be publickly whipp'd in Taunton, Glastonbury, Wells and Shepton Mallet, to be imprison'd for 6 months, and to find sureties for her good Behaviour, for as long a Time as the Justices at the next Quarter Sessions, shall think fit."—*Bristol Journal, Nov. 8th, 1746.*

**Medical Philology** (XL.).—In the *Promptorium* is "Enyn̄, or brynge forthe kyndelyngys. *Feto.*" Mr. Way's note says, "The verb to ean or yean, which is commonly applied only to the bringing forth of lambs, here appears to have had anciently the more general signification of the word from which it is derived, Ang.-Sax. eanian, *eniti, parturire.*"

"Eanlings" and "eaning-time" as used by Shylock (*Merchant of Venice*, I. iii. 80, 88) are familiar examples of the restricted meaning of the word.

In reference to the spelling ean or yean, Prof. Skeat's *Concise Etymological Dictionary*, ed. 1901, should be consulted.

"Kyndelyngys" in the *Promptorium* definition is of interest. Prof. Skeat gives (*op. cit.*) "kindel" as Middle English for progeny, from the Anglo-Saxon "cynd," nature. A recollection of this and of the German *kind*, child, makes Hamlet's description of himself as "a little more than kin, and less than kind" full of meaning when it is remembered that he uses it as a commentary on the title "son" given to him by his uncle-stepfather.

We have almost Englished the German "kindergarten."

**Clinical Records** (37).—Dr. George Fordyce, who came in 1762 from Edinburgh to London, very speedily made himself a name by a series of public lectures on medical science, which he afterwards published in a volume entitled *Elements of the Practice of Physic*, which passed through many editions. Unfortunately he was given to drink, and though he never was known to be dead drunk, yet he was often in a state which rendered him unfit for professional duties. One night when he was in such a condition, he was sent for to attend a lady of title who was very ill. He went, sat down, listened to her story, and felt her pulse. He found he was not up to his work; he lost his wits, and in a moment of forgetfulness, exclaimed: "Drunk, by Jove!" Still, he managed to write out a mild prescription. Early next morning he received a message from his noble patient to call on her at once. Dr. Fordyce felt very uncomfortable. The lady evidently intended to upbraid him either with an improper prescription or with his disgraceful condition. But, to his surprise and relief, she thanked him for his prompt compliance with her pressing summons, and then confessed that he had rightly diagnosed her case, that unfortunately, she occasionally indulged too freely in drink, but that she hoped he would preserve inviolable secrecy as to the condition he had found her in. Fordyce listened to her as grave as a judge, and said: "You may depend upon me, madam; I shall be as silent as the grave."—*Gentleman's Magazine.*

**Medical Scholarship.**—Dr. Irving H. Cameron, in his Presidential Address delivered before the Canadian Medical Association on the Overcrowding and the Decadence of Scholarship in the Profession, says: "That the literary side of the preliminary preparation for a medical education is nowadays too much neglected, and that a low level of scholarship obtains and is accepted, is abundantly evidenced by the characters of the answers given to examination questions, and by the questions themselves, by the applications and petitions of medical students to the various governing bodies, and by the formal announcements and kalendars of the Medical Colleges themselves. A comparison of the literary style of the medical text-books of to-day with that of the past generation enforces the same conclusions. Now this defect of scholarship is associated with, and to my mind, too oft begets defect of manners; and the courtesy and old-time courtliness of professional intercourse is rapidly disappearing. The pupil no longer greets his master with the outward evidences of profound respect, but commonly meets him on terms of equality. The practitioners of our art no longer manifest in their public intercourse and private conversation that deference which characterized the gentlemen of the old school. . . . Now I venture to group these two crying evils of our time—the over-crowding of the profession and its decadence in scholarship—together, because I believe the remedy for both is one and the same. Hear again what Mitchell Banks says upon the subject. . . . 'You can go on, no doubt, adding subject to subject, and examination to examination, but by so doing you only drive the student into further and further cramming. His serious defect at present is that, owing to the external cramming to which he is compelled to have recourse in order to master his subjects, he loses all power of thinking or reasoning for himself. He is being reduced to a mere grinding machine, which has to be well stoked up with scientific pabulum. . . . Well then, you ask, what is my remedy? My remedy consists simply in stiffening up the entrance examination.' . . . I am not one of those who think that a degree in science alone is the proper and essential precursor of entrance upon medical studies, but I hold strongly to my ancient faith in the *literae humaniores*—the basis of what was called in my younger days 'the education of an English gentleman'—as the proper substratum and foundation work upon which to build the superstructnre of a professional education. Such a course is particularly suited for the medical practitioner, doing away largely with the evils above complained of, exacting a higher degree of culture and refinement—thus diminishing the internal pressure of numbers, and by the establishment of a higher sense of professional character and conduct, effacing many of the crudities and barbarities which now obtain, for as Ovid long ago averred, 'Emollit mores nec sinit esse feros' . . . Having completed such a course with diligence and honesty, the new-born physician would enter upon his career of honourable usefulness with commingled modesty and confidence, and the evils which I have bemoaned at such length would cease in the land."—*The Montreal Medical Journal.*

